# The interaction between Shroom3 and Rho-kinase is required for neural tube morphogenesis in mice

**DOI:** 10.1242/bio.20147450

**Published:** 2014-08-29

**Authors:** Debamitra Das, Jenna K. Zalewski, Swarna Mohan, Timothy F. Plageman, Andrew P. VanDemark, Jeffrey D. Hildebrand

**Affiliations:** 1 Department of Biological Sciences, University of Pittsburgh, Pittsburgh, PA 15260, USA; 2 College of Optometry, The Ohio State University, Columbus, OH 43210, USA

**Keywords:** Shroom3, Rock, apical constriction, epithelial, neural tube

## Abstract

Shroom3 is an actin-associated regulator of cell morphology that is required for neural tube closure, formation of the lens placode, and gut morphogenesis in mice and has been linked to chronic kidney disease and directional heart looping in humans. Numerous studies have shown that Shroom3 likely regulates these developmental processes by directly binding to Rho-kinase and facilitating the assembly of apically positioned contractile actomyosin networks. We have characterized the molecular basis for the neural tube defects caused by an ENU-induced mutation that results in an arginine-to-cysteine amino acid substitution at position 1838 of mouse Shroom3. We show that this substitution has no effect on Shroom3 expression or localization but ablates Rock binding and renders Shroom3 non-functional for the ability to regulate cell morphology. Our results indicate that Rock is the major downstream effector of Shroom3 in the process of neural tube morphogenesis. Based on sequence conservation and biochemical analysis, we predict that the Shroom-Rock interaction is highly conserved across animal evolution and represents a signaling module that is utilized in a variety of biological processes.

## INTRODUCTION

The dynamic nature of the actin cytoskeleton is critical for regulating cellular processes and characteristics such as division, polarity, adhesion, migration, secretion and morphology (Babbin et al., 2009; Provenzano et al., 2009; Provenzano and Keely, 2009; Vicente-Manzanares et al., 2009a; Vicente-Manzanares et al., 2009b; Vicente-Manzanares et al., 2009c; Zhou et al., 2009; Sawyer et al., 2010; Sawyer et al., 2011; Allard and Mogilner, 2013; Etienne-Manneville, 2013; Flynn, 2013; Lagrue et al., 2013; Luo et al., 2013; Ojelade et al., 2013; Shirao and González-Billault, 2013). Through the use of genetic models systems and the mapping of mutations that cause human diseases, it has been well-established that errors in these processes underlie a wide range of maladies, including birth defects, cancer, kidney disease, and neuronal degeneration. One embryonic tissue that seems particularly sensitive to errors in cytoskeletal dynamics or architecture is the neural tube, the precursor to the brain and spinal chord (Campbell et al., 1986; Copp et al., 1990; Greene and Copp, 2009). While less common in the developed world, neural tube closure defects (NTDs), including spina bifida and excencephaly are among the most common birth defects and complicate approximately 1 in 1,000 births worldwide (Campbell et al., 1986; Copp et al., 1990). Despite an extensive amount of research, a clear understanding of the etiology of human NTDs has remained elusive (Greene and Copp, 2009; Greene et al., 2009b; Greene et al., 2009a).

To date, over 200 genes have been implicated in neural tube morphogenesis in mice. One of these genes is Shroom3, a member of a unique family of F-actin associated proteins that regulate cellular morphology during a wide range of developmental processes (Hildebrand and Soriano, 1999; Dietz et al., 2006; Hagens et al., 2006a; Yoder and Hildebrand, 2007; Bolinger et al., 2010). In vertebrates, the Shroom family is comprised of four members, Shroom1–4, each of which have been implicated in the regulation of various morphogenic events during embryonic development, including neural tube closure (Hildebrand and Soriano, 1999), remodeling of the vasculature (Farber et al., 2011), eye development (Fairbank et al., 2006; Plageman et al., 2010), gut morphogenesis (Grosse et al., 2011; Plageman et al., 2011a), neuronal architecture and function (Hagens et al., 2006b; Taylor et al., 2008), ENaC channel regulation (Assef et al., 2011), renal function (Köttgen et al., 2009), arterial hypertension (Sevilla-Pérez et al., 2008), and heterotaxy in humans (Tariq et al., 2011).

It is predicted that Shroom proteins function as adaptors that ultimately regulate the activity of contractile actomyosin networks. All Shroom proteins tested to date bind to both F-actin and Rho-associated kinase (Rock) via signature sequence motifs known as Shroom domain 1 (SD1) and 2 (SD2), respectively. In the case of Shroom3, this interaction with F-actin is required for its localization to the zonula adherens in polarized epithelial cells (Dietz et al., 2006). Shroom3 binds to Rock via its SD2 motif and recruits it to the zonula adherens (Nishimura and Takeichi, 2008). This results in localized activation of non-muscle myosin II (myosin II) via phosphorylation of myosin regulatory light chain (RLC) (Haigo et al., 2003; Hildebrand, 2005; Hagens et al., 2006b). As a result, the subcellular distribution of the actomyosin network within these cells is reorganized to form an apically positioned contractile ring. This ring exerts force to elicit apical constriction and facilitate the transition of columnar shaped cells into a wedge-shaped form (Hildebrand, 2005; Dietz et al., 2006). When this cell shape change occurs in a group of cells it can cause invagination or bending, leading to alterations in tissue morphology.

A new allele of Shroom3, S*hroom3^m1nisw^*, was recently identified in a forward genetic screen for ENU-induced mutations that cause neural tube defects (Zohn et al., 2005; Marean et al., 2011). Embryos homozygous for the S*hroom3^m1nisw^* allele exhibit exencephaly and phenocopy the gene trap allele of Shroom3, *Shroom3^gt(ROSA)53sor^*, suggesting that it is a functional null allele. In this study, we investigate the molecular basis for the loss-of-function phenotype associated with this allele. We show that this mutation abrogates the ability of Shroom3 to bind to Rock. This renders Shroom3 incapable of eliciting apical constriction via the activity of myosin II. These data indicate that the Shroom3-Rock interaction is vital for neural tube morphogenesis and that the majority of Shroom3 activity in apical constriction is mediated by Rock.

## RESULTS

### The *Shroom3^m1Nisw^* allele harbors a substitution mutation in the Shroom-domain 2 of Shroom3

**Fig. 1.**
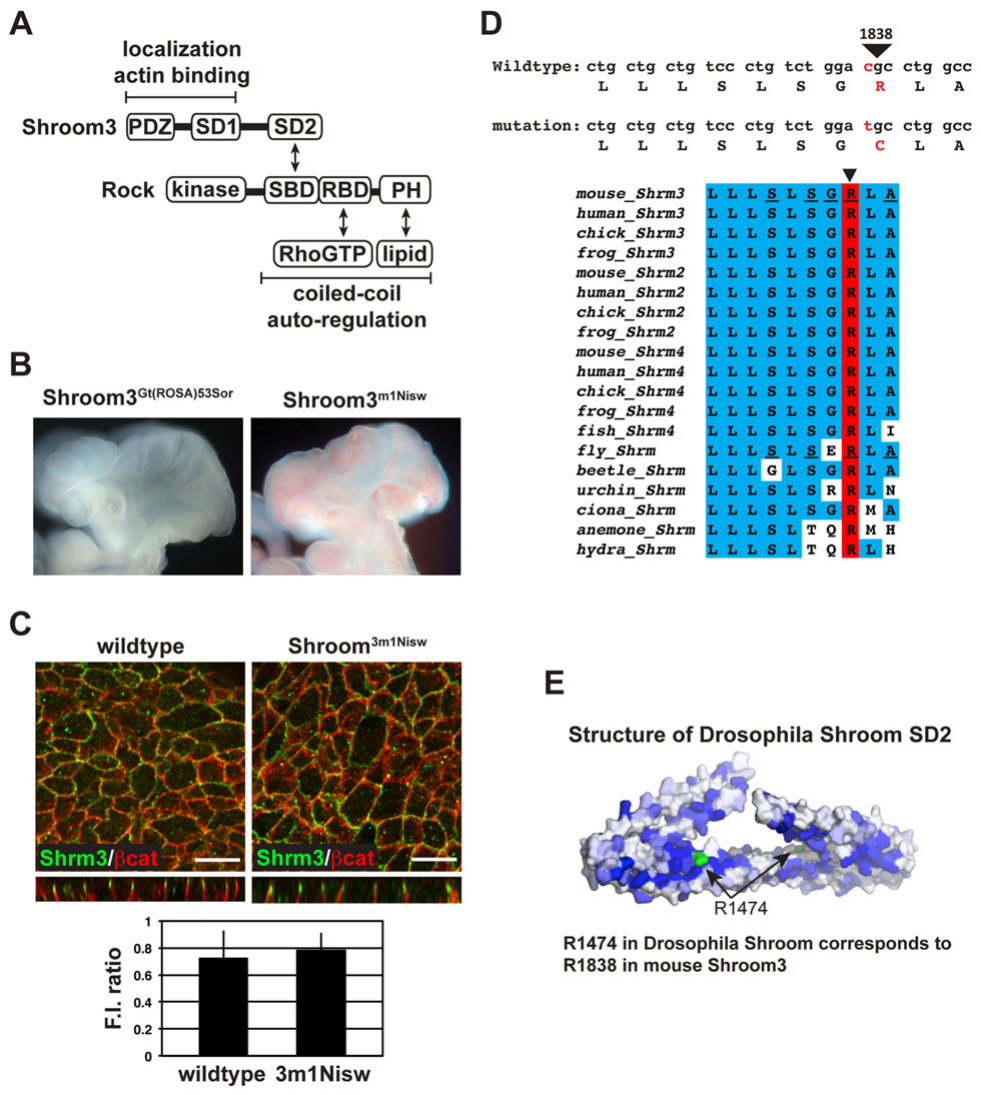
Arginine 1838 of Shroom3 is required for neural tube closure in mice but does not regulate protein expression or localization. (A) Schematic of the Shroom3-Rock signaling module. Arrows denote known direct interactions. SD, Shroom domain; PDZ, Psd-95/DlgA/ZO1 domain;. SBD, Shroom Binding domain of Rock; RBD, Rho binding domain; PH, pleckstrin homology domain. (B) Embryos homozygous for the Shroom3 null allele *Shroom3^Gt(ROSA)53Sor^* or the ENU allele *Shroom3^m1Nisw^* exhibit the same phenotype. (C) Expression of the Shroom3 R1838C protein. Wildtype or *Shroom3^m1Nisw^* homozygous e9.5 embryos were isolated, bisected sagittally to exposed the neural epithelium, stained in wholemount to detect Shroom3 (green) and β-catenin (red), and visualized by confocal microscopy. Z-projections are shown beneath; scale bar, 10 µm. Graph represents quantification of Shroom3 expression. Fluorescent intensity (F.I.) of Shroom3, expressed as the ratio of the average Shroom3 fluorescent intensity relative to the fluorescent intensity of β-catenin, from wildtype or *Shroom3^m1Nisw^* homozygous mutants. Error bars represent ± s.d., values are not significantly different using an unpaired *t*-test, n≥60 cells in two embryos per genotype; scale bar equals 10 µm. (D) The *Shroom3^m1Nisw^* mutation results in the substitution of a cysteine for a highly conserved arginine. Top panel shows the mutation while the bottom panel shows the sequence conservation of the SD2 in the vicinity of arginine 1838. Underlined amino acids constitute part of a conserved patch required for binding to Rock (Mohan et al., 2012). (E) Surface view of the Drosophila Shroom SD2 dimer as previous determined (Mohan et al., 2012) with the conserved arginine (R1474) residue in each monomer highlighted in green.

Shroom family proteins constitute a class of scaffolding protein that link the actin cytoskeleton to Rock localization via direct protein–protein interactions (Fig. 1A). One family member, Shroom3, has been shown to bind to Rock, recruit it to the zonula adherens, and facilitate the assembly of a circumapical contractile actomyosin network (Haigo et al., 2003; Hildebrand, 2005; Hagens et al., 2006b). Mice homozygous for a null allele of *Shroom3*, *Shroom3^gt(ROSA)53sor^*, exhibit severe neural tube defects (Hildebrand and Soriano, 1999). Recent studies identified an ENU-induced allele of *Shroom3* called *Shroom3^m1Nisw^* and embryos homozygous for this allele phenocopy homozygous *Shroom3^gt(ROSA)53sor^* embryos suggesting that this is a functional null allele (Fig. 1B) (Marean et al., 2011). Analysis of the *Shroom3^m1Nisw^* allele indicates a C-to-T missense mutation at nucleotide position 5744 in the *Shroom3* cDNA (accession number NM_015756), resulting in an arginine to cysteine amino acid substitution at position 1838 of Shroom3 (accession number NP_056571) (Fig. 1D). This would suggest that the mutant allele should still express full-length protein that is localized to the apical adhesion sites of cells. Consistent with this hypothesis, the staining of neural epithelium from wildtype or homozygous *Shroom3^m1Nisw^* embryos indicates that Shroom3 protein is expressed at approximately equal levels and exhibits similar subcellular distribution (Fig. 1C). Specifically, both the wildtype and mutant proteins are localized to the apical domain of adherens junctions in neural epithelial cells. To quantify the level of Shroom3 expression in these different genetic backgrounds, we measured the fluorescent intensity of Shroom3 relative to that of β-catenin. This analysis shows no significant difference between wildtype and mutant protein in either localization or expression. R1838 is located within the SD2 of Shroom3 and maps to a highly conserved patch of amino acids (Fig. 1D). We have previously shown that an SD2 variant harboring 5 substitutions within this patch (^1834^SLSGRLA^1840^ to ^1834^ALEADLE^1840^) abrogates the Shroom3-Rock interaction (Mohan et al., 2012). Importantly, an arginine is conserved at this position in all SD2 motifs identified to date, including the Drosophila Shroom protein, in which R1474 is analogous to R1838. We have recently solved the structure of the Drosophila Shroom SD2 motif (Mohan et al., 2012). In this structure, the SD2 forms a dimer and the two R1474 residues are surface exposed (Fig. 1E, R1474 residues are depicted in green). Based on this information, we predict that R1838 of Shroom3 is also surface exposed. As outlined in more detail below, this substitution mutation does not appear to affect the stability or folding of the protein. Therefore, these data indicate that the Shroom3 R1838C variant is defective for a specific activity or interaction that is required for neural tube closure in mice.

### Shroom3 R1838 is specifically required for binding to SBD of Rock

**Fig. 2.**
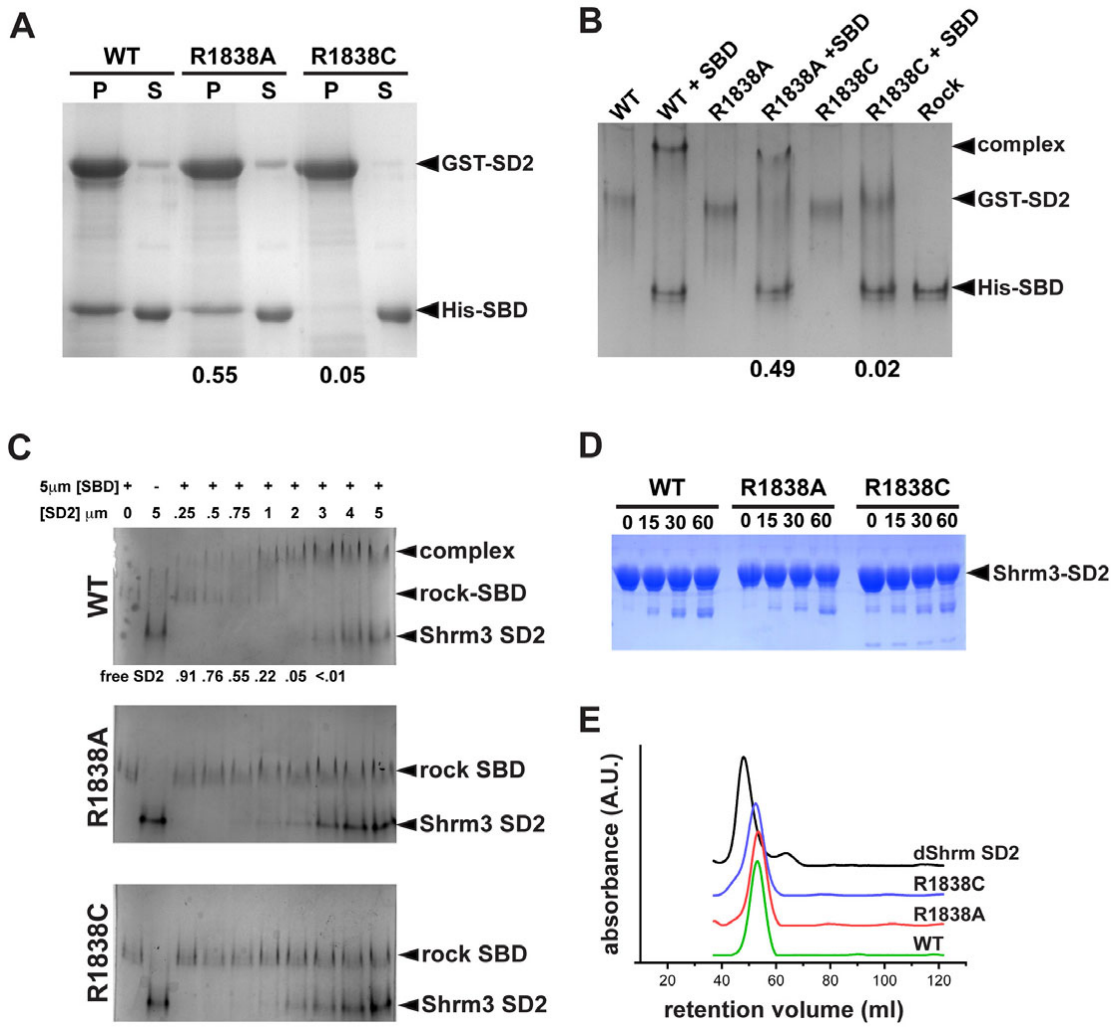
Arginine 1838 is required for the interaction between Shroom3 and Rock. (A) *In vitro* pull down assays using wild type and R1838 substitution variants of GST-Shroom3 SD2 (GST-SD2) bound to glutathione beads and His-tagged hRock SBD (His-SBD) in solution. The amount of Rock SBD in the pellet, relative to wild type, is indicated under the gel. (B) Gel mobility shift assay to detect the binding of GST-Shroom3 SD2 variants to the SBD of hRock1. Purified GST-Shroom3 SD2 and SBD proteins were mixed in solution, resolved on native PAGE gels, and detected by Coomassie Blue staining. The amount of complex formed relative to wild type is indicated beneath the gel. (C) Gel mobility shift assay to detect the interaction of untagged Shroom3 SD2 variants (Shroom3-SD2, amino acids 1642–1951) and untagged hRock1 SBD (Rock-SBD, amino acids 707–946). Increasing concentrations of SD2 proteins (indicated at top) were mixed with 5 µM SBD, resolved by native PAGE, and detected by Coomassie Blue staining. Values beneath the wild-type panel indicate the relative amount of free SBD. (D) Purified, untagged SD2 proteins were exposed to Subtilisin A for 0, 15, 30, or 60 minutes, resolved on SDS-PAGE gels, and stained with Coomassie Blue. (E) Size exclusion chromatography of purified, untagged Drosophila Shroom SD2 and the indicated mouse Shroom3 SD2 substitution variants.

Based on the above results and previous studies from our groups, we predicted that the Shroom3 R1838C protein is unable to interact with Rock. To test this hypothesis, we generated substitution variants R1838A and R1838C of mouse Shroom3 SD2 and tested their ability to bind to the Shroom-Binding domain (SBD) of human (h) Rock1 using *in vitro* binding assays. We generated two different substitution variants to also address the idea that an arginine residue is important at this position and that any alteration in Shroom3 function is not the result of the chemistry associated with a cysteine residue at this position. Alanine was selected because it is a small, non-polar, uncharged amino acid. First, we performed pull-down assays by mixing GST-Shroom3 SD2 variants bound to beads with soluble, His-tagged Rock1-SBD spanning amino acids 707–946. In this assay, relative to the wild-type SD2, the R1838A and R1838C variants exhibit an approximate 45% and 95% reduction, respectively, in the ability to bind the Rock SBD (Fig. 2A). To verify the results from the pull-down assay and assess the stability of the interaction, we mixed GST-Shroom3 SD2 with His-tagged SBD in solution and resolved the proteins by native gel electrophoresis. In this assay, wild-type GST-Shroom3 SD2 and the SBD form a stable complex that has reduced mobility in the native gel (Fig. 2B). In agreement with the pulldown assay, we found that the R1838C variant is incapable of forming a stable complex and essentially all of the SD2 and SBD proteins remain in the unbound state. In contrast, the R1838A exhibits an intermediate level of binding, with 51% of the GST-Shroom3 SD2 protein remaining unbound. These data suggest that the R1838 position is important for binding. However, because the alanine substitution results in an intermediate level of binding, it suggests that the cysteine mutation is more severe and that there may be some tolerance for different amino acids at this position. To further investigate this interaction and to verify that the GST moiety, because it is a dimer, did not influence binding, we assessed the ability of untagged SD2 and SBD proteins to form stable complexes using native gel shift assays (Fig. 2C). In these experiments, we can readily detect a complex consisting of wild-type SD2 and the Rock SBD. In contrast, we were unable to detect complex formation for the R1838A and R1838C variants, suggesting a significant decrease in their relative affinity for the Rock SBD.

Structural data for the SD2 from Drosophila Shroom suggests that the relevant residue in mouse Shroom3 should be surface exposed, however it is possible that the substitution mutations perturb the intramolecular interactions required for dimerization or alter protein folding and stability. The R1838C and R1838A variants exhibit the same mobility on a native gel (Fig. 2B,C), suggesting this is not the case. In addition, we compared the protease sensitivity of wild-type, R1838A, and R1838C SD2 proteins (Fig. 2D) and found no significant changes, indicating that the R1838 substitutions do not alter overall protein folding or stability. Finally, size-exclusion chromatography performed on untagged Drosophila Shroom SD2 and each of the mouse Shroom3 variants yielded similar profiles, indicating that the substitutions do not grossly alter the overall tertiary structure or promote the formation of protein aggregates (Fig. 2E). Taken together, these data indicate that the R1838A and R1838C proteins are virtually indistinguishable from the wild-type SD2 in folding and stability, suggesting that R1838 is playing a prominent role in mediating the Shroom-Rock interaction and not altering other aspects of SD2 structure.

### Shroom3 R1838A and R1838C fail to colocalize with Rock

**Fig. 3.**
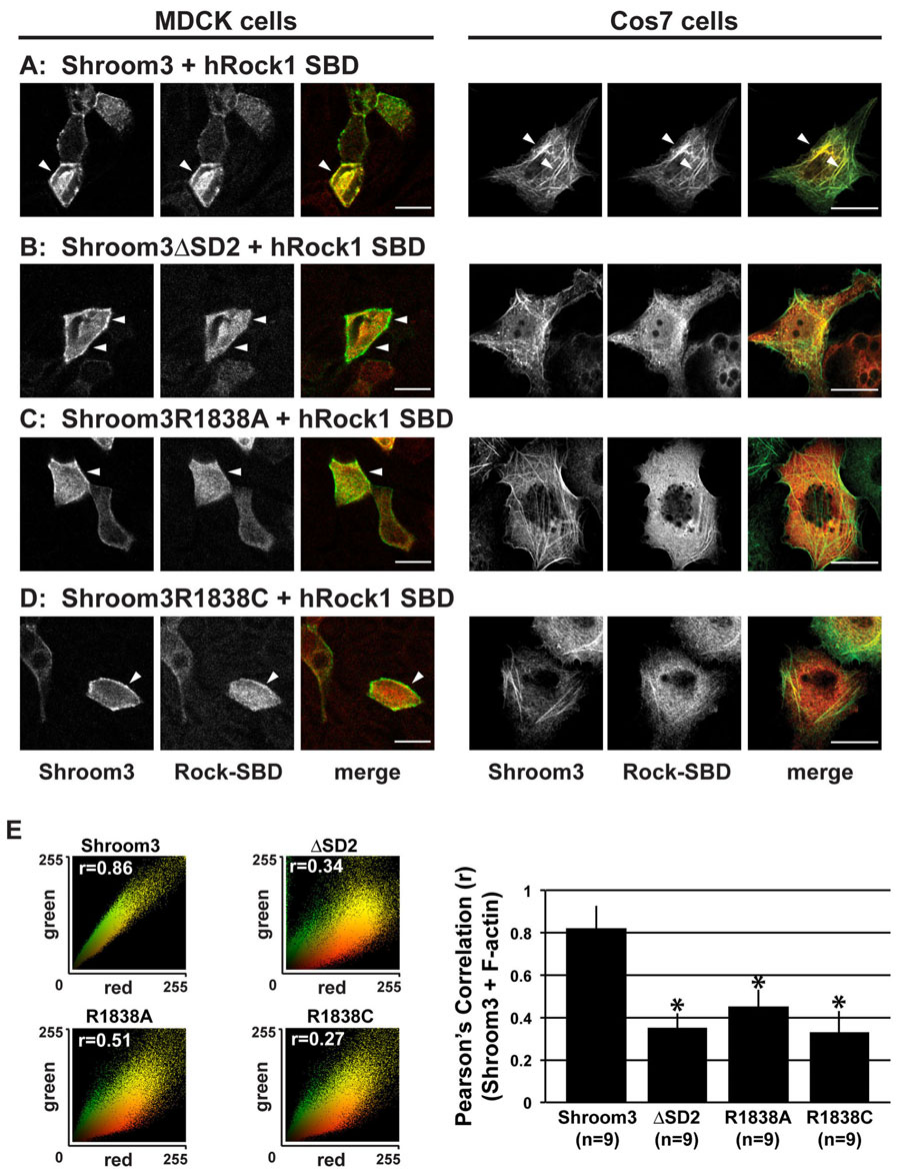
Shroom3 R1838 mutants fail to co-localize with the Rock SBD *in vivo*. (A–D) MDCK and Cos7 cells co-expressing the hRock1 SBD and either Shroom3 (A), Shroom3 ΔSD2 (B), Shroom3 R1838A (C) or Shroom3 R1838C (D) were grown on either transwell filters (MDCK cells) or fibronectin-coated coverslips (Cos7) and stained to detect Shroom3 (green) and the myc-tagged Rock SBD (red). Scale bar, 10 µm. (E) Quantification of colocalization. Left-hand panels are representative color scatter plots and indicate the degree of overlap between Shroom3 and the Rock-SBD with Pearson's correlation (*r* value) indicated in each scatter plot. Overlap was further quantified by plotting the average *r* (± s.d.) for the indicated number of cells in separate trials. * indicates *p*<0.01 using one-way ANOVA and Tukey HSD.

Shroom proteins bind directly to and recruit Rock to specific subcellular locales to regulate cell morphology and behavior (Dietz et al., 2006; Nishimura and Takeichi, 2008; Farber et al., 2011; Mohan et al., 2012). Based on the above data showing that Shroom3 variants R1838A and R1838C fail to bind Rock, we predicted that these mutants would also fail to recruit Rock to specific subcellular locales *in vivo*. To test this hypothesis, we co-expressed the Rock SBD with either wild type or substitution variants of Shroom3 in MDCK and Cos7 cells and assayed their co-localization. Shroom3 localizes to cell–cell junctions in MDCK cells and cortical actin and actin stress fibers in Cos7 cells, while the Rock SBD is typically cytoplasmic in these cells. If Shroom3 is capable of binding Rock and recruiting it, Rock will then colocalize with Shroom3. As expected, wild type Shroom3 and Rock colocalize to cell–cell junctions and actin stress fibers in MDCK cells and Cos7 cells, respectively (Fig. 3A). As a control, a version of Shroom3 lacking the SD2 (ΔSD2) is incapable of recruiting the SBD (Fig. 3B). Similar to the SD2 deletion variant, Shroom3 R1838A and R1838C mutants fail to colocalize with Rock in either MDCK or Cos7 cells (Fig. 3C,D). We performed colocalization analysis to quantify the degree of co-distribution between Shroom3 and the Rock SBD in Cos7 cells using color scatter plots (Fig. 3E, left panels). In these experiments, we see significant co-distribution of wildtype Shroom3 and Rock, while this linear relationship is greatly diminished in cells expressing the ΔSD2, R1838A, and R1838C variants. To further quantify these data, we plotted the Pearson's correlation (r value) for these scatter plots and we observe a significant difference between Shroom3 and the various SD2 variants (Fig. 3E, right panels). Thus, our data suggest that Shroom3-Rock binding is required for directing Rock to specific subcellular locales.

### Shroom3 R1838 is required for apical constriction and activation of the Rock-Myosin II pathway in polarized epithelial cells

**Fig. 4.**
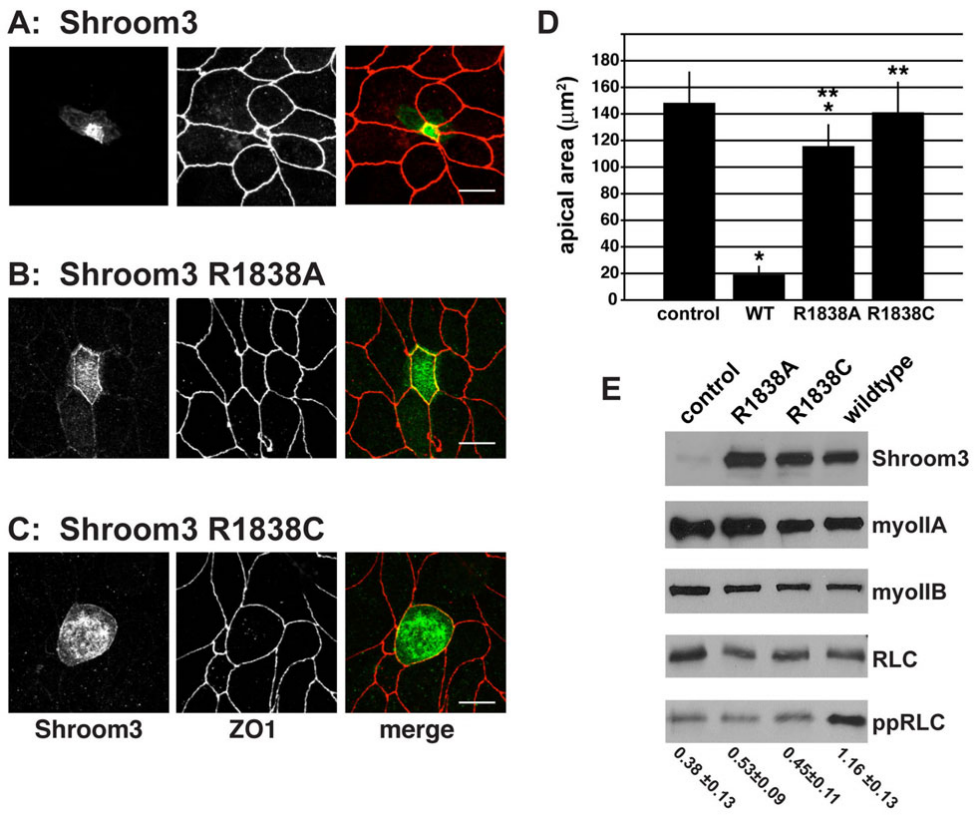
Shroom3 R1838 variants fail to induce apical constriction in MDCK cells. (A–C) MDCK cells transiently expressing Shroom3 (A), Shroom3 R1838A (B), or Shroom3 R1838C (C) were grown overnight on transwell filters and stained to detect Shroom3 (green) and the tight junction marker ZO-1 (red). Scale bar, 10 µm. (D) Quantification of apical constriction. Apical area was determined by measuring the area encircled by ZO1 staining of cells expressing the indicated Shroom3 protein. Error bars represent ± s.d. for at least 30 cells picked at random from three independent experiments, * indicates *p*<0.001 relative to untransfected control cells, ** indicates *p*<0.001 relative to cells expressing wildtype Shroom3 as determined by one-way Anova and Tukey HSD. (E) Lysates from cells expressing the indicated Shroom3 variant were probed by Western blot to detect Shroom3, myosin IIa, myosin IIb, RLC, and ppRLC. Representative blot is shown for ppRLC, values beneath each lane represent the average ppRLC:RLC ratio for three experiments (± s.d.) based on band intensity.

Previous work has shown that the Shroom3 SD2 is both necessary and sufficient to cause apical constriction in polarized MDCK cells and that this activity is dependent on Rock catalytic activity (Hildebrand, 2005; Dietz et al., 2006). To address whether alterations at R1838 prevent apical constriction, we expressed either wildtype or R1838 variants of Shroom3 in polarized MDCK cells and tested their ability to elicit apical constriction. MDCK cells expressing wild-type Shroom3 show dramatic apical constriction, demonstrating an 89% decrease in apical area relative to non-transfected cells (Fig. 4A,D). In contrast, both the R1838A and R1838C variants are significantly impaired in the ability to induce apical constriction in comparison to wildtype Shroom3 (Fig. 4B–D). Consistent with some of the *in vitro* binding data, the R1838A variant induces a small but significant degree of apical constriction; a 22% decrease in apical area relative to non-transfected cells (Fig. 4B,D). Cells expressing the Shroom3 R1838C variant exhibit only a slight, 8% decrease in apical area that is not significantly different from control cells (Fig. 4C,D). These data suggest that the R1838C has a more severe effect on apical constriction in MDCK cells in accordance with its inability to bind Rock. These results are likely due to the inability of these proteins to bind Rock and not the degree of protein expression or stability as all are expressed at similar levels (Fig. 4E).

To further characterize the R1838 variants and their ability to regulate actomyosin contractility, we assessed activation of Myosin II by measuring the phosphorylation of the RLC (Fig. 4E). Consistent with the above phenotypes, only cells expressing wildtype Shroom3 exhibit increased phosphorylation of RLC at Thr18/Ser19 as detected by Western blotting (Fig. 4E). We detect no change in the overall levels of myosin II or the RLC in cells expressing any of the Shroom3 proteins. The above results are consistent with the hypothesis that the interaction of Shroom3 and Rock directly correlates with the ability of Shroom3 to induce apical constriction.

**Fig. 5.**
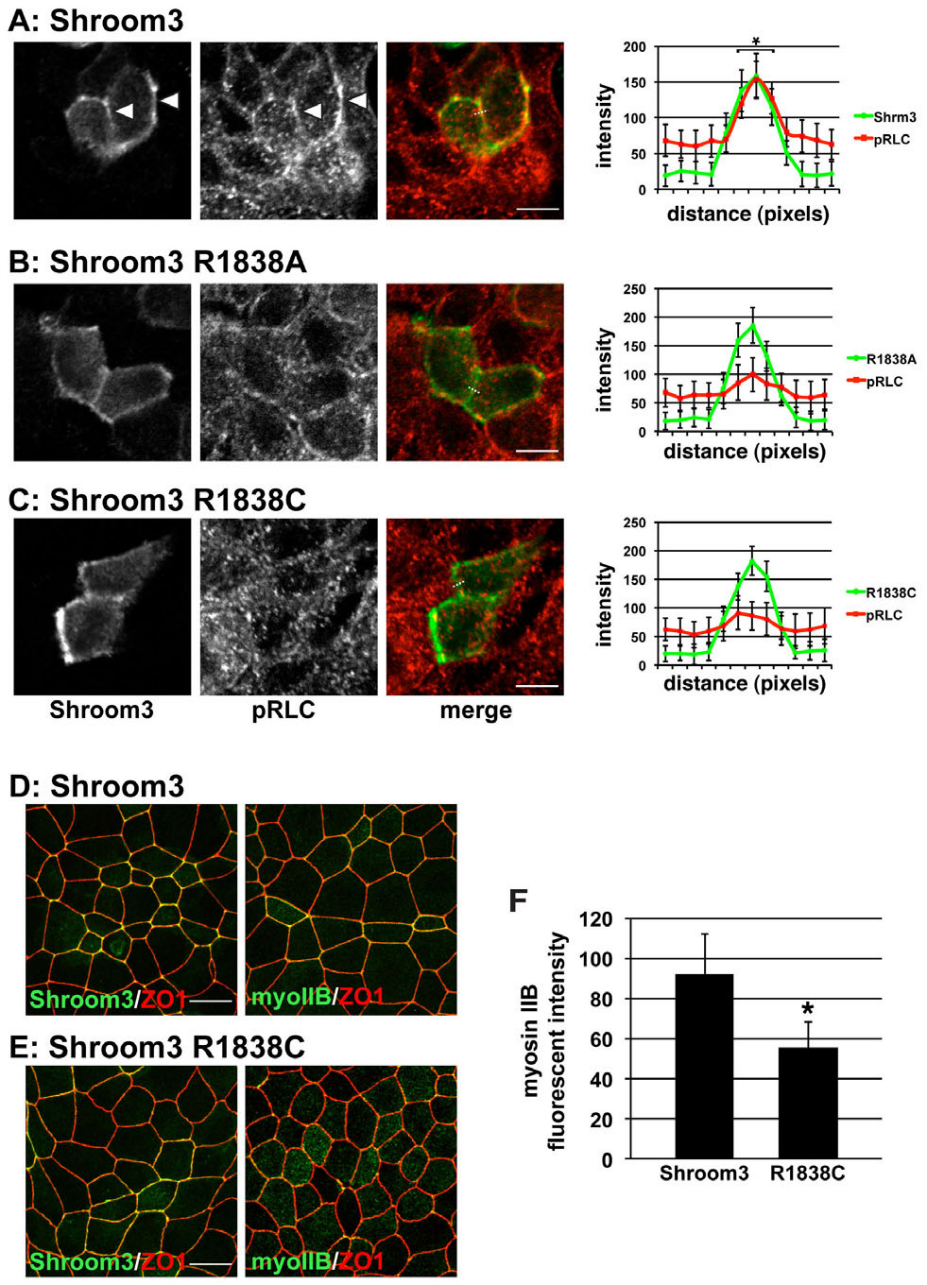
Shroom3 R1838 substitution mutations do not activate myosin II. (A–C) MDCK cells were transiently transfected with expression vectors for hRock1 and either Shroom3 (A), Shroom3 R1838A (B), or Shroom3 R1838C (C) and stained to detect Shroom3 (green) and phosphorylated RLC (pRLC, red). Right-hand panels show quantification of fluorescent intensity of Shroom3 (green) and pRLC (red) at the adherens junctions (arrowheads), as defined by Shroom3 staining. Dotted line denotes representative ROI used to measure fluorescent intensity; scale bar, 10 µm; * denotes significant differences (*p*<0.01 using an unpaired t-test) in pRLC fluorescence intensity in cells expressing wildtype Shroom3 relative to cells expressing either the R1838A or R1838C variant, n≧30 cells. (D,E) MDCK cells selected for expression of either Shroom3 or Shroom3 R1838C were stained to detect Shroom3 and ZO1 or myosin IIb and ZO1. Scale bar, 10 µm. (F) Quantification of myosin IIb localization in cells expressing Shroom3 or Shroom3 R1838C. Error bars indicate ± s.d., * indicates *p*<0.01 relative to Shroom3-expressing cells using an unpaired t-test, n≧30 cells in 3 experiments.

Under certain circumstances, the SD2 domain is both necessary and sufficient to induce changes in cytoskeletal organization and subsequent alterations in cell morphology (Hildebrand and Soriano, 1999; Hildebrand, 2005). SD2 elicits changes in cell morphology by altering the cellular distribution of contractile actomyosin networks (Hildebrand, 2005). This feat is accomplished via the direct association of the Shroom3 SD2 with Rock (Nishimura and Takeichi, 2008). We hypothesize that this interaction recruits Rock to specific subcellular compartments and activates it, resulting in the phosphorylation of Rock targets such as myosin RLC. This results in the activation of myosin II and the formation of a contractile actomyosin cable at zonula adherens which induces apical constriction (Hildebrand, 2005). Since Shroom3 R1838A and R1838C variants fail to bind Rock or cause apical constriction, we wanted to test if they are also incapable of activating Myosin II by assaying the phosphorylation status of RLC in polarized MDCK cells expressing these Shroom3 variants. MDCK cells were transfected with expression vectors for Shroom3 and Rock1, grown overnight on transwell filters to form polarized monolayers, and stained to detect Shroom3 and pRLC. Consistent with our previous findings, cells expressing Shroom3 R1838A or Shroom3 R1838C do not show enrichment of pRLC at cell junctions (Fig. 5B,C) compared to wild type Shroom3 (Fig. 5A). We quantified these data by plotting the fluorescence intensity of Shroom3 and pRLC. This was accomplished by drawing straight-line regions of interest (ROIs) that were 12 pixels in length and perpendicular to the zonula adherens of cells expressing the indicated Shroom3 protein. In cells expressing wildtype Shroom3, we see a significant increase in the fluorescence intensity of pRLC that co-distributes with Shroom3 at cell junctions relative to those expressing the R1838A or R1838C variant (Fig. 5A–C, right panels). Taken together, these results substantiate the role of this conserved arginine residue in mediating the Shroom3-Rock interaction and demonstrate that Rock binding, and subsequent localized activation of actomyosin, is required for proper neural tube morphogenesis.

To verify that R1838 is required for the assembly of apically located myosin II that causes apical contractility, MDCK cells expressing either wildtype Shroom3 or the R1838C variant were grown on transwell filters and stained to detect myosin IIb. Both the wildtype and R1838C proteins are localized to cell junctions (Fig. 5D,E). In cells expressing wildtype Shroom3 we observe a clear localization of myosin IIb to apical junctions, apically constricted cells, and straight cell junctions, consistent with cells under tension, as has been previously shown (Hildebrand, 2005). In contrast, cells expressing the R1838C variant exhibit neither recruitment of myosin IIb to apical junctions nor any changes in cell shape, suggesting that it is incapable of activating the Rock-Myosin pathway (Fig. 5D–F). Together, these results indicate that the R1838C variant of Shroom3 localizes correctly in polarized epithelial cells but is incapable of binding to Rock and subsequently cannot activate apical actomyosin contractility to facilitate neural tube closure.

## DISCUSSION

Neural tube closure is a complex morphogenetic event that has been intensely studied due to the severe birth defects associated with the failure of this process (Copp et al., 1990). Shroom3 plays an important role in neurulation in Xenopus, mouse, and chick embryos (Hildebrand and Soriano, 1999; Haigo et al., 2003; Nishimura and Takeichi, 2008). Shroom3 regulates tissue morphogenesis and cellular remodeling by modulating the cytoskeletal dynamics in the cell. Shroom3 binds to F-actin and Rock via signature sequence motifs and these interactions are required for *in vivo* activity. Importantly, it appears that most other Shroom family members function in an analogous manner to Shroom3 (Staub et al., 1992; Hildebrand and Soriano, 1999; Haigo et al., 2003; Hildebrand, 2005; Dietz et al., 2006; Fairbank et al., 2006; Hagens et al., 2006a; Yoder and Hildebrand, 2007; Farber et al., 2011). Thus, the Shroom-Rock pathway hints at a signaling module operating in a variety of cell types to regulate cellular behavior and morphology. However, the molecular mechanism of the Shroom-Rock interaction is still unknown. Our investigations into a substitution variant encoded by the *Shroom3^m1Nisw^* allele indicates that Rock binding is mediated by a defined part of the SD2 and subsequent apical recruitment is the vital step in Shroom3-mediated aspects of neural tube closure.

### R1838 is essential for Shroom3-induced changes in cell morphology

*Shroom3^m1Nisw^* mutant mice exhibit severe exencephaly that is attributed to a point mutation in the Shroom3 SD2 that changes arginine 1838 to cysteine. We have shown that the SD2 domain from Drosophila Shroom forms a three-segmented anti-parallel coiled-coil dimer with highly conserved surfaces that mediate Shroom-Rock interactions. (Mohan et al., 2012). One of these surface patches, ^1834^SLSGRLA^1840^, harbors R1838. We have shown that changing the positively charged arginine to either an uncharged polar amino acid (cysteine) or a nonpolar amino acid (alanine) disrupts binding to Rock. Interestingly, in some assays, these substitution variants have different severity, suggesting there is some tolerance at this position. However, both variants are significantly compromised in the ability to cause apical constriction, suggesting that an arginine at this position is essential. It will be interesting to solve the structure of the SD2-Rock complex to understand how this arginine residue participates in the interaction.

Using mouse as a model system, many labs have shown that the architecture and dynamics of the actin cytoskeleton must be precisely regulated during neural tube closure. This is demonstrated by the fact that mutations in cytsokeletal regulators such as Nap1 (Rakeman and Anderson, 2006), Abl1/2 (Koleske et al., 1998), p190RhoGap (Brouns et al., 2000), Mena/profilin (Lanier et al., 1999), Vinculin (Xu et al., 1998), NF1 (Lakkis et al., 1999), paladin (Roffers-Agarwal et al., 2012), Epb4.1l5 (Lee et al., 2007; Chu et al., 2013), and Marcks (Stumpo et al., 1995), all cause neural tube defects. We have previously shown that Shroom3 binds and bundles F-actin and may recruit Ena/Vasp proteins to the zonula adherens and that these activities are required for apical constriction of MDCK cells (Hildebrand and Soriano, 1999; Hildebrand, 2005; Plageman et al., 2010; Plageman et al., 2011b; Plageman et al., 2011a). Therefore, in addition to regulating localized actomyosin contractility, Shroom3 could also control aspects of actin dynamics to facilitate neural tube closure. However, our previous mapping studies have shown that F-actin binding is mediated by the SD1 motif, bounded by amino acids 754–1108, and is clearly distinct from R1838. Our work here also shows that mutation of R1838 does not perturb Shroom3 protein localization. Additionally, the putative Ena/Vasp binding motif (^1528^FPPPP) is also distinct from the SD2. Additionally, we have previously shown that Drosophila Shroom, which lacks a clear Ena/Vasp binding motif, still causes apical constriction in Drosophila embryos (Bolinger et al., 2010). Therefore, based on the molecular nature of the S*hroom3^m1nisw^* allele and our analysis here, we propose that these activities are likely to be intact in the R1838C substitution variant. Therefore, while this does not rule out the requirement of these other activities in Shroom3 function *in vivo*, it is clear that they are not sufficient for neural tube morphogenesis, while Rock binding is essential.

### The SD2-Rock interaction is evolutionarily conserved

In Drosophila, apically positioned contractile networks of actomyosin generate forces that are critical for germ band extension, ventral and dorsal closure, and various invaginations (Costa et al., 1994; Winter et al., 2001; Bertet et al., 2004; Nikolaidou and Barrett, 2004; Zallen and Wieschaus, 2004; Dawes-Hoang et al., 2005; Franke et al., 2005; Blankenship et al., 2006; Simões et al., 2006; Corrigall et al., 2007; Kolesnikov and Beckendorf, 2007; Mulinari et al., 2008; Xu et al., 2008). Our lab has shown that Drosophila Shroom, like Shroom3, binds to Rock and F-actin and induces robust apical constriction (Bolinger et al., 2010). We have solved the structure of the SD2 from dShroom and it is this analysis that allowed us to predict that R1838 of Shroom3 is surface exposed (Mohan et al., 2012). To verify that the Shroom-Rock interface is conserved, mutation of the analogous arginine in the dShroom SD2 (R1474 in dShroomA) renders the protein incapable of binding Rock (data not shown). Thus, we conclude that the Shroom-Rock-MyosinII pathway is evolutionarily conserved.

### Implications for understanding the Shroom-Rock interaction

An unanswered aspect of the Shroom-Rock pathway is if Shroom proteins are capable of directly activating Rock. It is predicted that, in its inactive state, Rock adopts a folded, autoinhibited conformation in which the C-terminal tail interacts with the N-terminal catalytic domain. It is thought that binding of proteins such as RhoA (in the GTP bound state) or lipids or caspase cleavage of the C-terminus relieves this intramolecular inhibition and activates Rock (Ishizaki et al., 1996; Leung et al., 1996; Matsui et al., 1996; Ishizaki et al., 1997; Amano et al., 1999; Feng et al., 1999; Araki et al., 2001; Coleman et al., 2001; Fukata et al., 2001; Sebbagh et al., 2001; Chen et al., 2002; Sebbagh et al., 2005; Yoneda et al., 2005). However, there is evidence for both Rho-dependent (Plageman et al., 2011b) and Rho-independent modes of Rock activation during Shroom3-induced apical constriction (Haigo et al., 2003; Hildebrand, 2005; Mohan et al., 2012). We predict that if Shroom binding is both necessary and sufficient for activation of Rock, then the Shroom3 R1838C substitution variant would be unable to perform either task.

Targeted Rock inhibition could serve as a potential therapeutic approach for many debilitating diseases, including cancer (Itoh et al., 1999; Kuzelová and Hrkal, 2008; Narumiya et al., 2009; Liu et al., 2011), obesity (Hara et al., 2011; Washida et al., 2011; Tokuyama et al., 2012), diabetes (Biswas et al., 2011), hypertension (Connolly and Aaronson, 2011), atherosclerosis (Zhou et al., 2011), and cardiovascular diseases (Dong et al., 2010). However, the Rock-Myosin II pathway is central to numerous cellular processes and signaling networks (Wei et al., 2001; Thumkeo et al., 2003; Shimizu et al., 2005; Thumkeo et al., 2005; Kamijo et al., 2011), suggesting that global inhibition of Rock function would likely disrupt multiple cellular processes with detrimental side effects. It would therefore prove helpful to understand the different mechanisms of Rock activation and its interaction with different downstream substrates in order to design methods for targeted modulation of Rock activity. Our structural studies into the regulation of Rock by Shroom proteins may provide new paradigms of Rock activation and novel insights into the treatment of diseases associated with dysregulation of Rock activity.

## MATERIALS AND METHODS

### Phenotypic analysis of mouse embryos

Mouse embryos from timed matings of heterozygous *Shroom3^gt(ROSA53)Sor^* or *Shroom3^m1Nisw^* mice were isolated at E10.5 dpc, fixed in 4% paraformaldehyde, and stored at 4°C. For detection of Shroom3, fixed embryos were cut sagittally along the midline to expose the neural epithelium, washed in PBT, blocked in PBT + 4% normal goat serum, and stained overnight at 4°C with anti-Shroom3 antibodies (UPT132, 1:100, (Hildebrand and Soriano, 1999; Hildebrand, 2005) and mAb anti-β-catenin (1:400, BD Transduction Labs, San Jose, CA, USA). Embryos were washed with PBT and primary antibody detected using Alexa 488-conjugated goat anti-rabbit secondary antibodies. Images were acquired using a Biorad Radiance 2000 Laser Scanning System mounted on a Nikon E800 microscope and processed using Photoshop. All mice were housed and cared for in accordance with guidelines established by the institutional care and use committees.

### Mutagenesis of Shroom proteins

Mouse Shroom3 SD2 mutants R1838A and R1838C were made using the QuikChange Site-Directed Mutagenesis Kit (Stratagene, Santa Clara, CA USA). Shroom3 mutagenesis was performed using the pCS2 vector harboring mouse Shroom3 (amino acids 286–1986) (Hildebrand and Soriano, 1999). The mutated Shroom SD2 sequences were further cloned from pCS2 into pGEX-3X or pET151 vectors for various biochemical assays and *in vitro* expression in E. coli CodonPlus RIPL cells. Recombinant proteins were expressed and purified as described (Farber et al., 2011; Mohan et al., 2012)

### Protein expression and purification

Large-scale protein expression of His-tagged SD2 and Rock SBD proteins was performed in BL21(DE3) E. coli cells using ZY autoinduction media as described (Farber et al., 2011; Mohan et al., 2012). The Shroom3 and Rock proteins were concentrated to 0.78 mg/ml (WT Shroom) and 1.43 mg/ml (R1838A) and 1.7 mg/ml (R1838C) and 1 mg/ml (WT Rock) in 20 mM Tris, pH 8.0, 0.5 M NaCl, 8% glycerol, and 5 mM dithiothreitol (DTT). For purification of GST-Shroom3 SD2 or small-scale (<50 ml) expression of His-tagged hRock SBD proteins, BL21 cells or RIPL cells harboring the relevant plasmids were induced with 0.5 mM isopropyl β-D-1-thioglactopyranoside (IPTG) for 2 hours and collected by centrifugation. Cells were lysed by sonication in NETN buffer (for GST-fusion proteins; 20 mM Tris, pH 8.0, 0.1 M NaCl, 1 mM EDTA, 0.5% NP-40) or His-lysis buffer (for His-fusion proteins; 20 mM Tris, pH 8.0, 0.5 M NaCl, 8% glycerol, 5 mM β-mercaptoethanol) supplemented with Protease inhibitor cocktail and soluble proteins were purified using either glutathione-sepharose resin or Ni-NTA beads. Beads were washed in lysis buffer and the proteins eluted with either free glutathione or imidazole in the respective lysis buffers. Drosophila Shroom SD2 (1393–1576) was purified as previously described (Mohan et al., 2012).

### Size exclusion chromatography

Untagged Drosophila Shroom SD2 (1393–1576), mouse Shroom3 SD2 (1642–1951), or mouse Shroom3 SD2 containing a R1838A or R1838C substitution were purified as described above and analyzed by size exclusion chromatography using a Sephacryl S-200 column. All of these runs were performed in 20 mM Tris pH 8.0, 250 mM NaCl, 2% glycerol, and 1 mM betamercaptoethanol. The flowrate of the column was 0.5 ml/min and the elution profile gathered by reading the absorbance at 280 nm.

### *In vitro* analysis of protein structure and function

GST pull-down assays were performed using either wild type GST-Shroom3 SD2 or R1844A and R1844C mutant versions (spanning amino acids 1562–1986) bound to Glutathione beads, and mixed with soluble, His tagged hRockI SBD (residues 707–946). The binding reaction was incubated for 2 hours at room temperature. Complexes were washed with NETN, resuspended in SDS-PAGE sample buffer, resolved on 12% SDS-PAGE, and detected using Coomassie Blue. For native gel electrophoresis, a fixed concentration (5 µM) of hRock SBD spanning amino acids 707–946 was mixed with increasing concentration of purified Shroom3 SD2 spanning amino acids 1642–1951 (0.25–5 µM) and incubated for 2 hours at 4°C. Samples were then loaded on 8% PAGE gels, resolved by electrophoresis at 4°C and proteins detected with Coomassie blue. For limited proteolysis studies, 50 µM of wild-type and mutant Shroom3 SD2 proteins were treated with 40 µg of the protease Subtilisin A for the indicated times and samples taken at each time point were resolved via SDS-PAGE. Purified WT and mutant proteins were concentrated to 1 mg/mL in buffer containing 2% glycerol, 250 mM NaCl, 20 mM Tris pH 8.0, and 1 mM β-mercaptoethanol. 500 uL were run over a Sephacryl S-200 gel filtration column, and traces were generated using Unicorn 6.3.2.89 Control Software.

### Cell culture and *in vivo* analysis

T23 MDCK cells were grown in EMEM supplemented with 10% FBS, pen/strep, and L-Glutamine at 37°C and 5% CO_2_. Cos7 cells were grown in DMEM supplemented with 10% FBS, pen/strep, and L-Glutamine under similar conditions. Cells were removed from the plates using Trypsin-EDTA and passaged every 2–3 days. For transient transfection of cells on transwell filters, cells were plated at a density of 8×10^5^ cells (MDCK) or 6×10^5^ cells per well (Cos7) and grown for 24 hours. Cells were transfected with the DNA of interest (1 µg) using Lipofectamine 2000 (Invitrogen, Grand Island, NY, USA) and grown for 24 hours prior to processing. For immunofluorescent analysis, cells were fixed using either −20°C methanol for 5 minutes or 4% paraformaldehyde (PFA) in PBS for 15 minutes. Fixed cells were stained with primary antibody for 1 hour at RT, washed in PBT three times for 5 minutes at room temperature, stained with secondary antibody for 1 hour at room temperature, washed as above and mounted using VectaShield (Vector Labs, Burlingame, CA, USA) or Immuno-fluore Mounting medium (MP Biomedicals, Santa Ana, CA, USA).

Shroom3-induced apical constriction using expression plasmids pCS2-Shroom3, pCS2-Shroom3 R1838A, pCS2-Shroom3 R1838C was performed and imaged as described previously (Hildebrand, 2005). Transfected cells were stained with primary antibodies UPT132 and Rat anti-ZO1 and detected with Alexa-488 or 568 conjugated secondary antibodies. Apical constriction was quantified by measuring the apical area of either parental or transfected cells, as determined by ZO1 staining, in ImageJ. To determine colocalizaton of Shroom3, Rock SBD, and pRLC, MDCK cells or Cos7 cells expressing Shroom3 variants and/or myc-tagged, wild type hRock1 SBD (spanning amino acids 681–942) were plated on either transwell membranes or fibronectin (Sigma, St. Louis, MO, USA.) coated coverslips, respectively, for 24 hours. To analyze role of Shroom3 on myosin IIb distribution, T23-MDCK cells were co-transfected with 20:1 ratio of linearized pCS2-Shroom3 or pCS2-Shroom3 R1838C with pTRE2-Hygro, selected in hygromycin, and surviving cells pooled and tested for Shroom3 expression. Cells were plated on transwell filters for 24 hrs and analyzed. Cells were stained to detect Shroom3, ZO1, myc-tag, ppRLC, myosin IIb, or F-actin (tritc-phalloidin). Primary antibodies were detected using Alexa-488 or 568 conjugated secondary antibodies and imaged as described above. Images were acquired using a Biorad Radiance 2000 Laser Scanning System mounted on a Nikon E800 microscope or Olympus Fluoview FV1000 Confocal microscope (FV10-ASW) with 40× oil objectives and processed using either ImageJ or Photoshop. To determine the degree of colocalization in Cos7 cells, ImageJ plug-ins Colocalization Finder and Mander's Coefficients were used to analyze individual channels from merged confocal images. To analyze the co-distribution of Shroom3 and pRLC, fluorescent intensity was measured along 12-pixel long line segments drawn perpendicular to the adherens junctions on individual channels. Data at each corresponding pixel from at least 30 measurements were averaged and plotted using Excel. To quantify fluorescent intensity of Myosin IIb at cells junctions using ImageJ, 1 pixel wide ROIs were drawn around individual cells using ZO1 as a guide. ROIs were copied to images of myosin IIb and fluorescent intensity was measured. To determine relative fluorescent intensity of Shroom3 proteins in neural epithelia, 1 pixel wide ROIs were drawn around individual cells based on Shroom3 or β-catenin staining. Fluorescent intensity was measured and the ration of Shroom3 to β-catenin staining determined. Measures of statistical significance were determined by two-tailed, unpaired, Student's *t*-Tests to distinguish significance between two data sets and a one-way ANOVA (Tukey's post-hoc) for comparison of 3 or greater data sets. For all graphs, error bars represent standard deviation (± s.d.). Western blots were performed as described (Farber et al., 2011).

### Antibodies used

Rabbit anti-Shroom3 UPT132 (1:100, Hildebrand, 2005), mouse anti-myc 9E10 (developed by J. M. Bishop, obtained from the Developmental Studies Hybridoma Bank, created by the NICHD of the NIH and maintained at The University of Iowa, Department of Biology, Iowa City, IA 52242), anti-RLC, p Ser19 RLC and ppThr18/Ser19 RLC (Cell Signaling, Danvers, MA, USA), anti-β-catenin (BD Transduction Lab), rabbit anti-nonmuscle Myosin IIa and IIb (Covance, Princeton, NJ, USA), and rat anti-ZO1 (R26.4C, Chemicon or Developmental Studies Hybridoma Bank, Iowa City, Iowa, USA). Primary antibodies were detected using Alexa-488 or 568 conjugated secondary goat anti-rabbit or goat anti-mouse (1:400, Invitrogen) or HRP-conjugated Goat anti-Rabbit or anti-mouse (GE Healthcare Bio-Sciences, Pittsburgh PA, USA).
